# Evaluating Therapy and Growth in Children with Phenylketonuria: A Retrospective Longitudinal Study from Two Romanian Centers

**DOI:** 10.3390/medicina60071185

**Published:** 2024-07-22

**Authors:** Meda-Ada Bugi, Iulius Jugănaru, Iulia-Elena Simina, Delia-Maria Nicoară, Lucian-Ioan Cristun, Giorgiana-Flavia Brad, Delia Huțanu, Raluca Isac, Kinga Kozma, Daniela Cîrnatu, Otilia Mărginean

**Affiliations:** 1 Ph.D. School Department, ‘Victor Babes’ University of Medicine and Pharmacy of Timisoara, 300041 Timisoara, Romania; bugi.ada@umft.ro (M.-A.B.);; 2 Department of Pediatrics I, Children’s Emergency Hospital ‘Louis Turcanu’, 300011 Timisoara, Romaniabrad.giorgiana@umft.ro (G.-F.B.); marginean.otilia@umft.ro (O.M.); 3 Department of Pharmacy, University of Medicine and Pharmacy ‘Vasile Goldis’, 310025 Arad, Romania; 4 Department XI Pediatrics, Discipline I Pediatrics, ‘Victor Babeş’ University of Medicine and Pharmacy of Timisoara, 300041 Timisoara, Romania; 5 Department of Research Center for Disturbances of Growth and Development in Children–BELIVE, ‘Victor Babeş’ University of Medicine and Pharmacy of Timisoara, 300011 Timisoara, Romania; 6 Department of Genetics, Center of Genomic Medicine, ‘Victor Babeş’ University of Medicine and Pharmacy, 300041 Timisoara, Romania; simina.iulia@umft.ro; 7 Department of Biology-Chemistry, Biology-Chemistry-Geography Faculty, West University of Timisoara, 300115 Timisoara, Romania; delia.hutanu@e-uvt.ro; 8 Department XI Pediatrics, Discipline III Pediatrics, ‘Victor Babeş’ University of Medicine and Pharmacy of Timisoara, 300041 Timisoara, Romania; isac.raluca@umft.ro; 9 Department of Preclinical Disciplines, Faculty of Medicine and Pharmacy, University of Oradea, 410000 Oradea, Romania; kkozma@uoradea.ro; 10 Regional Center of Medical Genetics Bihor, Emergency Clinical Hospital Bihor, 410000 Oradea, Romania; 11 Department of Medicine, University of Medicine and Pharmacy ‘Vasile Goldis’, 310025 Arad, Romania; 12 Romanian National Institute of Public Health, Regional Centre, 300230 Timisoara, Romania

**Keywords:** growth, therapy, protein intake, metabolic disease

## Abstract

*Background and Objectives*: Phenylketonuria (PKU) is a rare genetic disorder characterized by the inability to convert the essential amino acid phenylalanine into tyrosine. Early dietary treatment can successfully prevent complications, but controversies still exist regarding the attainment of normal growth in these patients. *Materials and Methods*: Eighteen patients with PKU from two Romanian reference centers were compared to eighteen non-PKU controls, matched for age and gender. The comparisons used weight-for-height, weight-for-age, height/length-for-age, and body mass index-for-age z-scores from birth to three years of age. *Results*: The PKU study group consisted of nine boys and nine girls, with a median follow-up period of thirty-six months (interquartile range = 9.75). While median values of all four growth metrics remained within the normal range across the entire study period, weight-for-age z-scores were significantly lower in PKU patients throughout most of the study (*p* < 0.001). *Conclusions*: The persistent lower weight-for-age z-scores of the PKU patients compared to controls indicate that ongoing monitoring and potential adjustments in dietary therapy may be necessary to further optimize growth outcomes.

## 1. Introduction

Phenylketonuria (PKU, MIM 261600) results from a deficiency in the hepatic enzyme phenylalanine hydroxylase (PAH; EC 1.14.16.1; OMIM 612349). It is a rare condition, which occurs in approximately 1 in 24,000 individuals, affecting an estimated 450,000 people globally [1]. This autosomal recessive metabolic disorder is characterized by the inability to convert the essential amino acid phenylalanine (Phe) into tyrosine, resulting in the accumulation of toxic metabolites [2]. Many countries, including Romania, implement routine newborn screening programs for early detection of PKU [3]. These screening programs facilitate the early diagnosis of PKU in infants, allowing for the commencement of standard care protocols, ideally within the first ten days of life [4]. Still, it remains unclear how many European centers can meet this target [5]. Advancements in newborn screening programs, combined with effective dietary interventions, have significantly extended the lifespan of individuals diagnosed with PKU [6].

The prevalence and severity of PKU vary across different populations. Individuals with severe PAH deficiency, known as classical PKU, exhibit untreated plasma Phe levels exceeding 1200 μmol/L. Those with a less severe phenotype, including moderate PKU and mild PKU, have untreated plasma Phe levels ranging from 600 to 1200 μmol/L. Patients with plasma Phe levels below 360 μmol/L are classified as having mild hyperphenylalaninemia (mHPA). Target Phe levels for all these patients are 120–360 μmol/L [4,7,8].

The goal of PKU treatment remains to prevent cognitive impairment, promote normal growth, and maintain good health and quality of life into adulthood [2,9]. Classical PKU treatment involves adherence to a specialized diet [10], which includes stringent restriction of phenylalanine intake by limiting dietary protein, supplemented with a Phe-free formula (PFF), amino acid formula (AAF), and low-Phe glycomacropeptide (GMP), all of them providing essential amino acids, vitamins, and other nutrients [11,12]. At approximately six months of age, a more concentrated protein substitute needs to be introduced [13]. This process complements the challenge of transitioning from liquid formula to solid foods and accepting the AAF [14] with its intense flavor [14,15]. During infancy, protein substitutes provide 50–80% of the total protein requirements [5,16,17]. Infants with classical PKU require a greater total protein intake than healthy populations due to inefficient absorption and use of amino acids from AAF sources [17,18]. Medical therapy mainly consists of the synthetic form of tetrahydrobiopterin (BH4), known as Sapropterin. PKU patients who respond to treatment demonstrate the capacity to increase their dietary Phe intake [19,20,21] by at least two-fold while maintaining plasma Phe levels within the normal range. Moreover, a subset of patients can maintain plasma Phe levels within the target range without any restricted diet. The transition to a more typical diet has been suggested to provide substantial benefits, including improved nutritional status and enhanced quality of life for these patients [10,22]. The milder form of PKU requires no dietary restrictions [10] or allows for very high tolerance [23,24].

Managing the diet of PKU patients requires a nuanced approach, balancing the need for adequate nutrient intake to support growth and development with strict limits on intact protein to maintain optimal blood phenylalanine levels [25]. The extent to which this process influences growth is still a matter of controversy, with studies across the world documenting different results. Recent reports have indicated no significant difference in growth and body composition between PKU patients and normal individuals [26,27,28]. Conversely, other studies show that in the first three years of life, PKU patients have shorter length/height z-scores [12,29,30,31,32,33,34,35] and a lower weight-for-age z-score, while birth weight was comparable to healthy controls [29,33,34,35]. Research on growth in mHPA patients reveals that their weight-for-age z-score, height-for-age z-score, length-for-age z-score, and body-mass-index-for-age z-score were generally similar to the reference population [33,36,37]. However, Thiele et al. [34] observed significantly lower height-for-age z-scores in mHPA patients compared to healthy children during the first six years.

Considering that no official information is available on growth in Romanian PKU patients, the present paper aimed to evaluate the growth trajectories of children with PKU from 2 Romanian centers over a period of 36 months.

## 2. Materials and Methods

### 2.1. Study Protocol

In this retrospective longitudinal study, we analyzed the growth of children with PKU from two Romanian pediatric referral centers for inherited metabolic disorders (Timisoara and Oradea) from January 2010 to March 2024. Inclusion criteria were as follows: age between one month to three years old; a diagnosis of PAH deficiency confirmed biochemically (elevated Phe blood levels, a Phe/Tyrosine (Tyr) ratio above 3) and through molecular studies (identification of two pathogenic variants in PAH); maintenance of continuous Phe-restricted diet, supplemented by Phe-free substitutes and specially produced low-protein foods, and regular attendance at scheduled clinical check-ups. Patients with major congenital anomalies, additional chronic disorders, or incomplete data were excluded. A total of 18 children met these criteria: 14 from the Children’s Emergency Hospital ‘Louis Turcanu’ Timisoara, a tertiary center specializing in the care of patients with inborn errors of metabolism from southwestern Romania, and 4 children from the Emergency Clinical Hospital, Bihor. To facilitate comparison, we included a control group consisting of an equal number of non-PKU children matched by age and gender who were admitted to the Pediatric Ward of the Children’s Emergency Hospital ‘Louis Turcanu’. The exclusion criteria for control subjects consisted of known diagnoses that could influence growth, such as malnutrition, growth hormone deficiency, genetic disorders, and acute or chronic gastrointestinal diseases. The research adhered to the guidelines from the Declaration of Helsinki. The research protocol of the study obtained approval from the Ethics Committees of both ‘Louis Turcanu’ Hospital (No. 20181/11 December 2023) and the Emergency Clinical Hospital (No. 2171/7 June 2024). Informed consent was provided by the parents of all subjects.

### 2.2. Data Collection

Retrospective data consisting of demographics (age and gender), genotype, growth progress, Phe blood levels, and dietary intake information were recorded from diagnosis until three years of age.

#### 2.2.1. Anthropometric Measurements

Growth measurements included weight and height from several time points (birth, the moment of diagnosis, and quarterly during the first three years) as part of the routine pediatric evaluation by the Romanian PKU guidelines. All measurements were performed by qualified health professionals, including pediatricians, dietitians, or specialized nurses, who adhered to standard procedures. Height measurements were taken using a calibrated ASTRA stadiometer with a precision of 0.1 cm, while weight measurements were obtained using a digital ASTRA weighing scale with an accuracy of 100 g. For infants up to 12 months, a manual infant scale and measuring board were used to measure recumbent length, transitioning to a manual scale and standing height measurements after that. Measurements were obtained during scheduled clinical consultations, with three measurements obtained at each visit and their average considered. Body mass index (BMI) was calculated as weight (kg)/height (m^2^). The child’s age, sex, weight, length, and height measurements were used to determine the following growth indicators: weight-for-age z-score (WAZ), length/height-for-age z-score (HAZ), and body mass index (BMI)-for-age z-score (BAZ). We investigated growth patterns in relation to the intake of protein and energy during the first three years of life. This period was chosen because previous studies had shown that this was the period in which the growth restriction was most evident [30]. BAZ values below −2 or between +1 and +2 were considered underweight and overweight, respectively, while values between −2 and +1 were considered normal weight. HAZ values below −2 or above +2 were classified as short stature and tall stature, respectively, while values between −2 and +2 were regarded as normal stature [38].

#### 2.2.2. Phe Blood Measurements

Blood phenylalanine concentrations using the Fluoro-Immuno-Enzymatic method technique were regularly assessed to evaluate the efficacy of the prescribed diet for each patient and to monitor adherence to the treatment regimen. Patients were sampled at the moment of diagnosis, weekly from birth to 1 year, fortnightly from 1 year to 2 years, and monthly from 2 years onward. For the study, we used the value from diagnosis and the quarterly averages from the prior three months for all later measurements. Patients were managed in accordance with European guidelines for PKU, aiming to maintain recommended target blood phenylalanine levels below 120–360 μmol/L for individuals aged 0–12 years. The guidelines state no intervention is required if the untreated blood Phe concentration is less than 360 μmol/L. Treatment is recommended up to age 12 if the untreated Phe is between 360 and 600 μmol/L, while lifelong treatment is recommended if the untreated Phe is above 600 μmol/L [4,8].

Good metabolic control for children below seven years was defined as blood Phe levels below 242 μmol/L [4]. Patients were classified into four phenotypic categories: classic, moderate, and mild PKU and mHPA, based on Phe levels measured at diagnosis [4,7,8].

#### 2.2.3. Dietary Intake

Phe-restricted diets, supplemented with Phe-free amino acid mixtures and customized low-protein foods, were initiated promptly after diagnosis [33]. Caloric and protein intake were evaluated using a 3-day 24 h dietary recall [39], analyzed by an experienced dietitian at each center. Based on these records, daily intake of phenylalanine, total protein, and calories from both protein substitutes and natural foods were computed for each individual. When these records were unavailable, self-reported dietary records were used instead. The dietary protein intake in grams per kilogram per day was compared with the safe levels recommended by the WHO [40]. The protein-to-energy ratio (P:E ratio) was computed for each dietary recall and expressed as grams of protein per 100 kilocalories. Significantly, consumption of protein-rich foods, such as meats, fish, eggs, dairy products, legumes, and moderately proteinaceous staples, such as cereals, was restricted [41]. The prescribed daily natural protein intake was dynamically adjusted according to age and recent blood Phe measurements. Furthermore, the recommended daily provision of protein substitutes was delineated by age, following the Romanian PKU treatment protocol principles, with patients advised to divide these allocations into at least four servings daily.

The data extracted from the medical records encompassed an overview of dietary interventions, the extent of Phe restriction, types of Phe-free/low-Phe protein substitutes, allocation of Phe from vegetables, fruits, or unrestricted sources [42], and dietary Phe tolerance, quantified as the total daily intake of Phe in milligrams per day (mg/d) [43].

In the context of this study, a liberalized diet refers to a diet that allows unrestricted consumption of dietary protein [4], prescribed only to patients with mHPA or responsive to Sapropterine.

### 2.3. Use of OpenAI ChatGPT-3.5

The article utilized the ChatGPT-3.5 model, developed by OpenAI, for language and grammar checks. The authors rigorously reviewed, edited, and refined the results, assuming full responsibility for the content presented in the publication.

### 2.4. Statistical Analyses

Analyses were performed using IBM SPSS Statistics for Windows 20 (IBM Corporation, Armonk, NY, USA), JASP (Version 0.18.1, Amsterdam, The Netherlands), and GraphPad Prism software (Version 10.2.3, San Diego, CA, USA). The normality of data distribution was evaluated using the analytical Shapiro–Wilk test. Descriptive summaries of continuous variables included the number of observations, the percentages for categorical variables, mean and standard deviation for continuous variables with normal distribution, and median and interquartile range (IQR) for continuous variables with abnormal distribution. Demographic and growth parameters of PKU patients were compared to the control group using the Mann–Whitney test, while box plots were used to visualize the results of growth trajectories. The chi-squared test and Fisher’s exact test were used, as appropriate, to compare categorical variables, such as gender, mother’s education level, breastfeeding status, diagnosis type, PKU type, and diet type. The dynamic assessment of the four indicators—HAZ, WAZ, WHZ, and BAZ—was conducted using the Individual Assessment Module of the Anthro application. Developed by the World Health Organization, Anthro monitors children’s growth and nutritional status from birth to 5 years old [38,44]. We used Spearman’s rank correlation coefficient (ρ) to examine the relationship between growth metrics, Phe blood levels, and dietary parameters. The significance level for all analyses was considered at *p* < 0.05.

## 3. Results

### 3.1. General Characteristics of the Study Population

Overall, the study included 18 patients with PKU who were followed longitudinally and had diet records in each of the four quarters of the first three years of life. They were recruited from 2 specialist PKU centers: the Children’s Emergency Hospital, Timisoara (*n* = 14), and the Emergency Clinical Hospital, Bihor (*n* = 4). The control group consisted of 18 age- and gender-matched controls selected from apparently healthy children.

Phenylketonuria patients were reviewed by the same dietitian at each center. There were 9 boys and 9 girls, with a median follow-up period of 36 months (IQR = 9.75). General characteristics are shown in Table 1.

For almost all patients, diagnosis of PKU was performed by means of newborn screening using the Guthrie test, with only one patient being diagnosed late. The median age at diagnosis was 21 days (minimum 4 and maximum 390 days), and all children began their diet within 24 h of receiving the diagnosis. Most of the patients (72.2%) were diagnosed with classical PKU (eight males and five females), one female patient with moderate PKU, and four patients with mHPA (one male and three females).

In PKU patients, the mean birth weight was 3184 ± 527 g (females, 2889 ± 479 g; males, 3430 ± 395 g), all having a normal birth weight (2500–4000 g). The mean body length was 49.4 ± 2.1 cm (females, 48.6 ± 2.5 cm; males, 50.2 ± 0.8 cm). Compared to controls, the birth height was borderline significantly lower (*p* = 0.04), while the birth weight was similar. During infancy, the assessment of the feeding type revealed that breastmilk was a source of natural protein in 83.3% of infants.

Fourteen children were treated with a Phe-restricted diet under the supervision of the same (original) dietary team to keep their Phe blood levels below 242 mmol/L (4 mg/dL), while four children were on a liberalized diet.

### 3.2. Dietary Intake

The protein intake varied from 1.3 to 3.22 (mean 2.17 ± 0.36) g/kg per day, representing 90–292% (mean 175.9 ± 43.1%) of the Recommended Dietary Allowance (RDA). The caloric intake varied from 71.9 to 116.7 (mean 91.5 ± 10.3) kcal/kg per day, representing 90–292% (mean 175.9 ± 43.1%) of the RDA. These values are above the RDAs, and at the same time, higher than the recommended safe intake according to the World Health Organization. Table 2 presents a summary of the dietary intake of patients during the study period.

**Figure 1:**
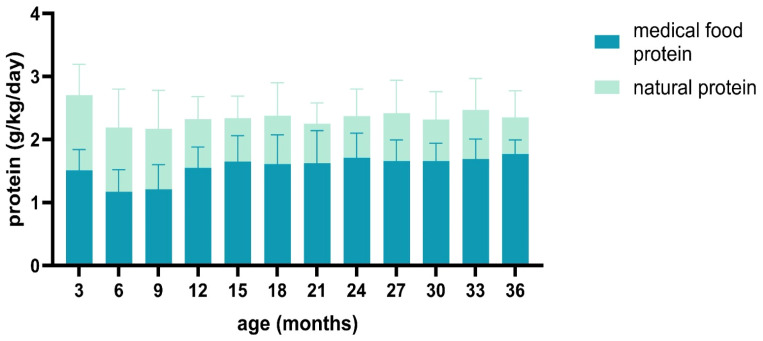
Distribution of protein sources across total protein intake.

Additionally, 72% (71.8 ± 12.53) of the protein consumed by PKU patients was from medical food, while 28% (27.6 ± 11) was from natural food. The quantity of medical food (Phe-free formula until six months and amino acid-based formula after six months) was lower during the first year. Still, it increased and stabilized afterward, as shown in Figure 1.

The median Phe concentrations during the entire study period were used to assess metabolic control. Good metabolic control was defined as below 242 μmol/L. Globally, 15 patients (83.3%) had good metabolic control (Supplementary Table S1).

### 3.3. Changes in Anthropometric Characteristics

**Figure 2:**
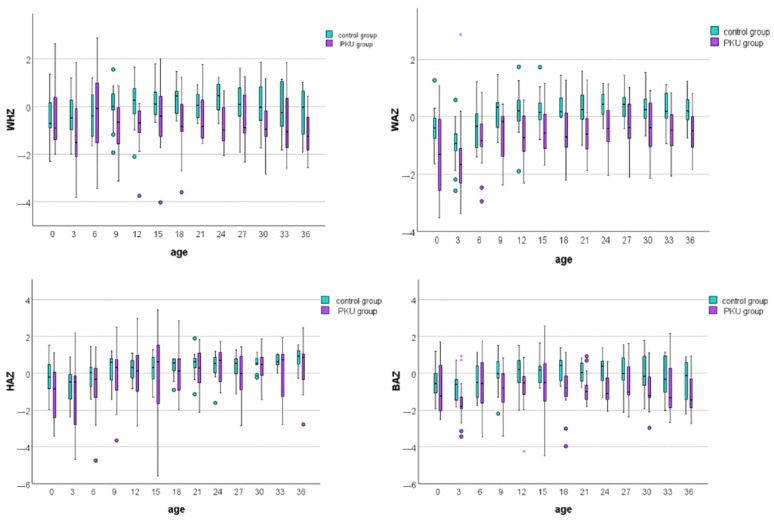
Box plot comparison of weight-for-height, weight-for-age, height-for-age, and BMI-for-age z-scores between PKU patients and controls. Each box plot displays the median (central line), interquartile range (box), and whiskers, which extend to the most extreme data points within 1.5 times the interquartile range from the first and third quartiles. Circles (○) represent mild outliers (1.5–3 times the interquartile range, IQR), while asterisks (*) indicate extreme outliers (beyond 3 times the IQR).

We compared the growth trajectories of the PKU group with Romanian growth standards by calculating z-scores for weight, height, and BMI for both PKU patients and controls (Figure 2). The most significant differences were found in weight-for-age z-scores, indicating that PKU patients weighed less than controls at nearly every age assessment (Figure 2; Supplementary Table S2). Height remained similar between the two groups over three years, except at birth. Moreover, despite children with PKU having smaller WHZ and BAZ values than controls at different age points, all median z-score values at each age fell within the range of −2 to +1.

According to the BMI-for-age z-score and length/height-for-age z-score, most PKU patients had normal weight and height during the entire study period, as seen in Table 3.

**Figure 3:**
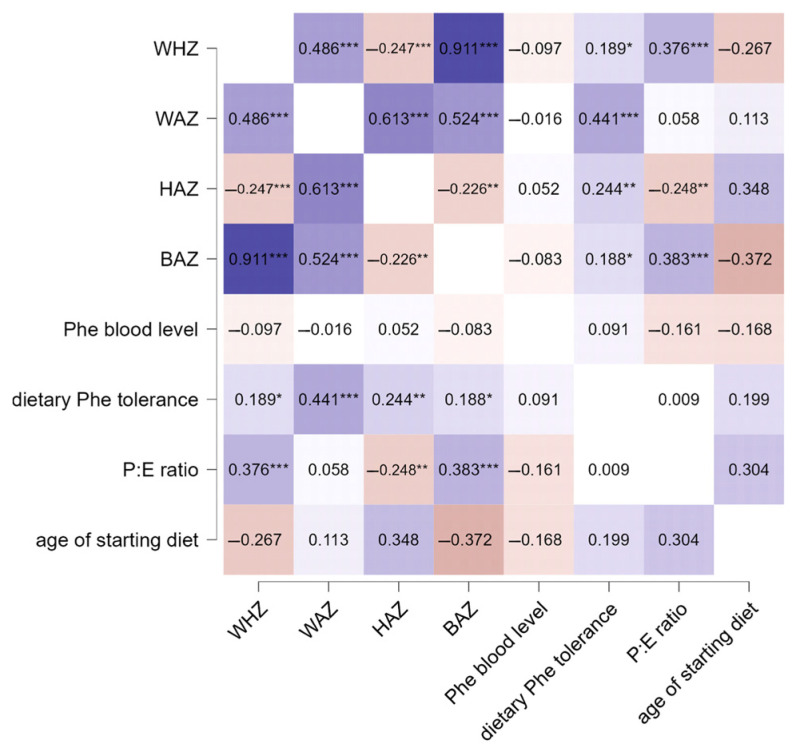
Correlation analysis between growth, Phe blood levels, and dietary parameters in PKU patients. Significance levels were *** *p* < 0.001, ** *p* < 0.01, and * *p* < 0.05. The color gradient in the figure indicates the level of significance, where darker shades denote stronger associations and lighter shades indicate relatively lower statistical significance.

Finally, Spearman correlation analysis was performed to evaluate possible correlations between growth parameters, blood Phe levels, and dietary parameters. The results showed that WAZ, HAZ, and BAZ correlated positively with dietary Phe tolerance (ρ = 0.441, ρ = 0.244, and ρ = 0.188, respectively), while WHZ, HAZ, and BAZ also correlated with the P:E ratio (ρ = 0.376, ρ = −0.248, and ρ = 0.383). The results are summarized in Figure 3.

## 4. Discussion

Phenylketonuria is a genetically determined metabolic disease, where secondary prophylactic measures effectively prevent disease complications [45,46,47]. In the beginning, the only therapeutic option for children diagnosed with classical PKU was medical nutritional therapy [48,49]. Early initiation, within the first days after birth, of a phenylalanine-restricted diet supplemented with medical food substantially optimized the neurological and cognitive outcomes of patients with PKU [50]. Dietary management, primarily structured around control of protein consumption [24], involves transitioning from natural protein sources, such as human milk and those derived from plant foods, fruits, and vegetables [51], to a semi-synthetic diet, where a substantial amount of protein is derived from an amino acid mixture that lacks phenylalanine [37]. Medical formulas exhibit slight variations in nutritional compositions across different countries. For instance, many European formulas lack added fat or fiber, formulas containing glycomacropeptide (GMP) often have higher carbohydrate and energy content [52], some amino acid mixtures include added oligosaccharides, and formulas designed for the first year of life contain more carbohydrates and fat [53,54]. In Romania, there are currently two options each for PFF and AAF. The PFF options have standard dilution but differ in nutritional content: one has 466 kcal/100 g, 50.1 g of carbohydrates, 23 g of lipids, and 0.61 g of L-alanine, while the other has 508 kcal/100 g, 54 g of carbohydrates, 28 g of lipids, and 0.5 g of L-alanine. For AAF, one option provides 60 g of protein equivalent with 12 g of carbohydrates, and the other offers 74 g of protein equivalent without carbohydrates. Until recently, these formulas were only available in powdered form, whereas now they also come in single-dose packaging. In other countries, to facilitate acceptance, they are also available in granule form [55]. Regarding GMP products, which were accepted in the Romanian Guideline in 2022, they are recommended for children over 3, so they have not been used during the studied period. Although the ideal timeframe for initiating dietary treatment is within 7–10 days after birth [4], delayed treatment initiation is still common in Romania, typically occurring within a few weeks and only occasionally within mere days postpartum. These delays often stem from various factors, including late dispatch of samples from maternity wards, logistical challenges in sample transportation to laboratories, and laboratory processing and reporting of elevated blood phenylalanine levels. As a result, the authorized personnel responsible for notifying families and directing them to the nearest treatment center face significant challenges in ensuring timely intervention.

However, dietary management presents a considerable challenge for patients and their caregivers, often constraining adherence [56,57,58]. Besides dietary therapy, two medical treatments are available, the most commonly used being Tetrahydrobiopterin (BH4) or Sapropterin. These oral medications act as a cofactor for the PAH protein, enhancing its activity in a subset of patients [59]. They can increase dietary phenylalanine tolerance in individuals with milder forms of PKU, thereby potentially allowing for a more liberalized diet [10]. The other treatment option is Pegvaliase [60], an enzyme that catabolizes phenylalanine and is available only for patients over 16. Additionally, messenger RNA therapeutics delivered via lipid nanoparticles hold promise for treating metabolic diseases caused by protein deficiency, including phenylketonuria [61].

Earlier studies conducted between 1980 and 1990 in children with PKU, regardless of treatment status, displayed subnormal growth [62]. As dietary management practices improved, the inadequate growth observed in treated children led to changes in dietary prescriptions. Consequently, many studies from various countries now report adequate physical growth in these children [63]. Still, the subject of optimal growth in phenylketonuria is part of an ongoing debate, with controversial findings still existing concerning the influences of PKU nutritional therapy on the physical development of these children.

The reduced growth rate observed in some studies does not appear to have an endocrinological etiology, as both the somatotropic and thyrotropic axes function normally [2]. It is more likely influenced by therapeutic interventions and genetic predisposition [2,45]. Most studies have reported no correlation between Phe concentrations and PKU patients’ growth [2,11,12,31,33,35,64]. Our study’s findings aligned with this pattern, as none of the evaluated anthropometric indexes correlated with blood Phe levels. The effect of total protein intake, natural protein, and protein substitutes has also been evaluated regarding growth, with inconsistent results [65]. Suboptimal growth was revealed by earlier studies, in which total protein intake was equivalent to the RDA for the general population [30,31,65]. This prompted the European PKU guidelines to recommend a higher protein intake, advising an additional 40% of protein from phenylalanine-free amino acids beyond the WHO safe levels [40] to compensate for the inefficient utilization of L-amino acids, the decreased absorption of protein fractions, and to help lower blood phenylalanine concentrations [16,66,67]. The increase in total protein intake was subsequently associated with satisfactory growth in most studies [19,26,35,36,37,68,69,70,71], with a few exceptions [2,31,33,72]. In the present study, total protein intake ranged from 1.3 to 3.22 g/kg per day (mean 2.17 ± 0.36 g/kg per day), which corresponds to an average of 175.9 ± 43.1% of the recommended daily allowance, similar to the mean total protein intakes prescribed in Western Europe [34,40]. Children in both groups exceeded the safe levels of protein intake (mean PKU 194%, range 141–251%; mean control 188%, range 133–272%) [17]. Moreover, 72% of the protein consumed by our PKU patients originated from medical food, similar to a previous study by López-Mejía et al. [73]. Research studies have also examined how various fractions of total protein affect growth. However, they have not conclusively linked anthropometric measurements with natural protein intake [2,24,34] or protein substitute intake [12,24,33]. Furthermore, evidence from the literature regarding the effects of BH4 treatment on growth has demonstrated inconsistent results [25,73,74,75]. Singh et al. observed a significant increase in height z-score after two years of administering BH4 therapy [25]. In contrast, Aldámiz-Echevarría et al. reported growth impairment in patients on a Phe-restricted diet alone and those on BH4 therapy [75]. Similarly, Daly et al. found no difference in growth velocity between patients on BH4 therapy and those on conventional PKU treatment [76]. The latter two studies had longer observation periods of five and three years, respectively. In our study, four patients (P9 and P16–18) with mHPA underwent BH-4 therapy and adhered to a liberalized diet. They displayed notably lower mean growth metrics for the entire study period than controls: WHZ −0.98 vs. −0.035, WAZ −1.23 vs. 0.09, HAZ −0.38 vs. 0.46, and BAZ −1.1 vs. −0.08 (*p* < 0.001). These differences were statistically significant despite falling within the normal range. The impact of disease phenotype has been a subject of debate. Some studies have indicated normal growth regardless of PKU phenotype [37], whereas others [29,33,34] have observed suboptimal growth in children with a severe phenotype. Patients with classical PKU from our study also presented with lower mean growth metrics for the entire study period compared to controls: WHZ of −0.76 (−1.49, 0.20), WAZ of −0.63 (−1.21, 0.14), HAZ of 0.11 (−1.02, 1.08), and BAZ of −1 (−1.7, 0.11). These differences were all statistically significant (*p* < 0.001) despite falling within the normal growth range.

Regarding the influence of demographic characteristics, certain studies have reported delayed catch-up growth in height and head circumference among boys [31,53], and a tendency toward overweight in females older than eight years [33]. Also, studies have shown that poor adherence to treatment can result from limited access to care or inadequate social support, financial constraints, lower educational attainment, and diminished self-efficacy [77]. Patients in our study showed a spectrum of caregiver educational levels (% of mothers with university education) and financial resources, with all having equal access to treatment.

Regarding birth anthropometrics, most studies reported normal birth weight [29,33,34,36,37] and height [32,33,36,37], likely due to the maternal liver’s metabolism of phenylalanine through phenylalanine hydroxylase [73]. However, there are also reports of smaller birth height within the PKU population [12,30,31,34]. The mean birth weight of our PKU patients was similar to those of the control group, whereas birth weight showed a borderline significant decrease (*p* = 0.04). This is consistent with the findings of Verkerk et al. [30] and Dhondt et al. [32].

Analyzing the growth trajectories of our two study groups, we observed that PKU patients weighed less than controls throughout the study period. Despite growth trajectories within normal ranges for most PKU patients in our study group, their weight-for-age consistently remained lower than the controls, consistent with previous studies [12,29,33,34]. Overall, the percentage of eutrophic anthropometric measurements was high (76.3%), similar to previous reports [78].

Although birth height was lower in children with PKU, complete catch-up occurred and was maintained during the entire study period, with similar values recorded by both study groups. This overall normal physical development during the first years of life is in accordance with some of the previous findings [24,79], but not with all. Ilgaz et al. reported impaired growth during the first years of life [65]. Also, several other studies reported shorter length/height in early childhood [29,30,32,33,34,35]. Variations in reported growth outcomes across the studies may reflect differences in dietary protocols, rates of patient adherence to diet, access to specific products, the extent of micronutrient supplementation [79], and phenotype distribution [6].

The results obtained from this study have several important prospects for practical application and further research. Firstly, they provide a valuable reference point for healthcare providers in Romania and potentially other similar settings to assess and compare the growth trajectories of children with PKU. By demonstrating that current dietary management practices are generally effective in supporting normal growth, these findings can reassure clinicians and caregivers about the efficacy of existing interventions. Moreover, the identification of persistent lower weight-for-age z-scores suggests specific areas where dietary therapy might be improved and can guide the development of more tailored nutritional plans aimed at addressing weight deficits, thereby enhancing overall growth outcomes. The study’s findings also underscore the need for continuous monitoring and potential adjustments in dietary practices, which could be incorporated into clinical guidelines and standard care protocols for PKU management.

Several limitations should be acknowledged. First, the retrospective nature of the study resulted in varying degrees of data completeness. Additionally, this retrospective design introduces the possibility of selection bias, as the study only included children with phenylketonuria who attended check-ups, potentially affecting the generalizability of the findings. Self-reported data were used in a few cases, which also could be a source of bias. Moreover, the three-year follow-up period may not capture long-term growth trends, as other studies suggest that significant weight gain can occur [73,80,81,82,83,84], especially in girls after eight years of age [33]. The small sample size further limited our ability to draw definitive conclusions, extrapolate findings, and examine the effects of genetics or socioeconomic status on the growth of these children. Additionally, our controls originated from children admitted to the hospital for routine check-ups or minor ailments, with no suspected or known diagnoses that could influence growth, rather than from a general population sample. Lastly, we were unable to assess body composition or collect data on micronutrients and trace elements. Future studies should aim to include a larger and more diverse sample, along with a prospective design to better capture the long-term effects of dietary management on growth.

## 5. Conclusions

In summary, this study provides the first insights into the growth trajectories of Romanian children with PKU during early childhood, demonstrating adequate growth across a diverse population that includes both classical PKU and mHPA, with overall good metabolic control. These findings highlighted the effectiveness of current dietary management practices in supporting normal growth in children with PKU. However, the persistent lower weight-for-age z-scores compared to controls indicated that ongoing monitoring and potential adjustments in dietary therapy may be necessary to further optimize growth outcomes. Future prospective, multicenter studies involving a larger cohort of Romanian children are warranted to validate these findings and explore additional factors influencing growth and development in this population.

## Figures and Tables

**Table 1:** Demographical and clinical characteristics.

Variables	PKU Group	Control Group	*p*-Value
Current age (months)	29.7 ± 10.2	32 ± 7.4	0.461
Males *n* (%)	9 (50)	9 (50)	1
Mother’s education level *n* (%)			
university	9 (50)		
professional school	4 (22)		
high school	5 (27.8)		
Breastfed *n* (%)	15 (83.3)		
Birth weight (grams)	3184 ± 527	3297 ± 381	0.327
Birth height (cm)	49.4 ± 2.1	50.6 ± 1.2	0.04
Diagnosis type *n* (%)			
NBS	17 (94.4)		
late diagnosis	1 (5.6)		
Untreated Phe level µmol/L	1280 (542, 1816)		
PKU type *n* (%)			
classical	13 (72.2)		
moderate	1 (5.6)		
mild	4 (22.2)		
Diet type *n* (%)			
restricted	14 (77.8)		
unrestricted	4 (22.2)		

Abbreviations: PKU, phenylketonuria; NBS, newborn screening; Phe, phenylalanine. Data are expressed as mean and standard deviation or median and IQR, accordingly.

**Table 2:** Mean nutritional and metabolic parameters according to age.

Age (Months)	Total Protein g/kg/day	Natural Protein g/kg/day	AAF g/kg/day	Dietary Phe Tolerance mg/day	kcal /kg/day	Glucides /kg/day	Lipids /kg/day	P:E Ratio
3	2.55 ± 0.38	1.19 ± 0.49	1.51 ± 0.33	264 ± 118	103 ± 6.8	16.6 ± 1.34	3.16 ± 0.25	2.48 ± 0.37
6	2.02 ± 0.33	1.02 ± 0.61	1.17 ± 0.35	245 ± 43	90.8 ± 10.8	14.7 ± 2.2	2.81 ± 0.41	2.30 ± 0.36
9	2.04 ± 0.24	0.96 ± 0.61	1.21 ± 0.39	273 ± 75	91 ± 9.9	14.7 ± 1.94	2.81 ± 0.37	2.30 ± 0.36
12	2.23 ± 0.24	0.77 ± 0.36	1.55 ± 0.33	296 ± 90	88.8 ± 10.6	14.1 ± 2.04	2.68 ± 0.38	2.58 ± 0.51
15	2.26 ± 0.34	0.69 ± 0.35	1.65 ± 0.41	292 ± 109	87.6 ± 9.1	13.8 ± 1.78	2.64 ± 0.34	2.63 ± 0.57
18	2.19 ± 0.34	0.77 ± 0.52	1.61 ± 0.46	301 ± 107	82.1 ± 22.8	13 ± 4.25	2.47 ± 0.81	2.66 (2.2, 3)
21	2.17 ± 0.46	0.63 ± 0.33	1.62 ± 0.52	274 ± 130	87.5 ± 9.69	13.9 ± 1.85	2.64 ± 0.35	2.53 ± 0.65
24	2.22 ± 0.31	0.66 ± 0.43	1.71 ± 0.39	294 ± 123	90.4 ± 10.9	14.4 ± 2.08	2.75 ± 0.39	2.41 ± 0.54
27	2.08 ± 0.36	0.76 ± 0.52	1.66 ± 0.33	292 ± 131	90.7 ± 10.3	14.3 ± 1.92	2.72 ± 0.36	2.39 ± 0.55
30	2.05 ± 0.32	0.66 ± 0.44	1.66 ± 0.28	307 ± 121	89.6 ± 8.9	14.4 ± 1.88	2.73 ± 0.35	2.36 ± 0.53
33	2.07 ± 0.35	0.78 ± 0.50	1.69 ± 0.32	303 ± 130	92.8 ± 8.5	14.7 ± 1.91	2.80 ± 0.36	2.33 ± 0.49
36	2.08 ± 0.33	0.58 ± 0.42	1.77 ± 0.22	270 ± 69	94.1 ± 11.2	15.2 ± 2.21	2.88 ± 0.42	2.27 ± 0.49

Abbreviations: AAF, amino-acid-based formula; Phe, phenylalanine; P:E ratio, protein/energy ratio. Data are represented as mean ± SD and median (IQR), as appropriate.

**Table 3:** Anthropometric status of PKU patients by age group.

Age	Underweight	Normal Weight	Overweight	Stunted	Normal Height	Tall Stature
0–12 months	2 (11.7)	15 (88.3)	0	2 (11.7)	12 (70.6)	3 (17.7)
13–24 months	1 (6.6)	14 (93.4)	0	2 (13.3)	10 (66.7)	3 (20)
25–36 months	2 (15)	9 (70)	2 (15)	2 (15.3)	9 (69.2)	2 (15.4)

Data are represented as number of observations (percentage).

## Data Availability

The original contributions presented in the study are included in the article. Further inquiries can be directed to the corresponding author.

## Supplementary Materials

The following supporting information can be downloaded at: https://www.mdpi.com/article/10.3390/medicina60071185/s1, Table S1: Patient Genetic Profiles and Phenylalanine Levels in PKU; Table S2: *p*-values for age-based comparison of growth parameters between PKU and control group.
