# Supplementary material for: Evaluating Therapy and Growth in Children with Phenylketonuria: A Retrospective Longitudinal Study from Two Romanian Centers
Source: Medicina (Kaunas). 2024 Jul 22;60(7):1185. doi: 10.3390/medicina60071185 (PMC11279053; doi:10.3390/medicina60071185)
Supplement: Supplementary file 1 [file medicina-60-01185-s001.zip › medicina-3101802-supplementary/Supplementary Table S1.pdf]

Table S1. Patient Genetic Profiles and Phenylalanine Levels in PKU

Patient	mutation	zygosity status	untreated Phe blood level (μmol/L)	mean Phe blood level (μmol/L)
P1	NM_000277.3:c.472C>T<br>NP_000268.1:p.Arg158Trp<br>NM_000277.3:c.1222C>T<br>NP_000268.1: p.Arg408Trp	heterozygous	1816.1	141.3 (124, 196.6)
P2	NM_000277.3:c.1222C>T<br>NP_000268.1: p.Arg408Trp	homozygous	1513	197 (115.7, 247.2)
P3	NM_000277.3:c.1222C>T<br>NP_000268.1:p.Arg408Trp	homozygous	1816	156.5 (131.8, 197.7)
P4	NM_000277.3:c.1066-11G>A<br>NM_000277.3:c.1222C>T<br>NP_000268.1:p.(Arg408Trp	heterozygous	378	157 (133.1, 193.7)
P5	NM_000277.3:c.754C>T<br>NP_000268.1: p.Arg252Trp<br>NM_000277.3:c.782G>A<br>NP_000268.1:p.Arg261 GLN	heterozygous	1816.1	150.6 (89.6, 166.1)
P6	NM_000277.3:c.1222C>T<br>NP_000268.1:p.Arg408Trp<br>NM_000277.3:c.782G>A<br>NP_000268.1:p.Arg261Gln	heterozygous	1816.1	201.9 (174.6, 333.5)
P7	NM_000277.3:c.1222C>T<br>NP_000268.1:p.(Arg408Trp)	homozygous	2360	193.7 (139.2, 1213.7)
P8	N/A	N/A	1749	479.5 (145.9, 649.8)
P9	NM_000277.3:c.1315+1G>A<br>NM_000277.3:c.533A>G<br>NP_000268.1:p.Glu178Gly	heterozygous	393.4	245.1 (210.4, 272.4)
P10	NM_000277.3:c.1222C>T<br>NP_000268.1:p.(Arg408Trp)	homozygous	670.7	191.9 (123, 217.8)
P11	NM_000277.3:c.1222C>T<br>NP_000268.1:p.(Arg408Trp)	homozygous	1228.8	216.5 (186.1, 331.1)
P12	NM_000277.3:c.1222C>T<br>NP_000268.1:p.(Arg408Trp)	homozygous	617.6	181.6 (133.1, 211.3)
P13	NM_000277.3:c.1222C>T<br>NP_000268.1:p.(Arg408Trp)	homozygous	1331.7	272.4 (195.2, 301.1)
P14	NM_000277.3:c.1222C>T<br>NP_000268.1:p.(Arg408Trp)	homozygous	1816.1	164.5 (125.5, 267.8)
P15	NM_000277.3:c.1222C>T<br>NP_000268.1:p.(Arg408Trp)	homozygous	715.5	148.3 (105.1, 199.8)
P16	NM_000317.3:c.84-3C>G	homozygous	367	205.8 (146.7, 277.6)
P17	NM_000277.3:c.1208C>T<br>NP_000268.1:p.(Arg403Val)<br>NM_000277.3:c.842G>A	heterozygous	227	186.4 ± 3
P18	NM_000277.3:c.1222C>T<br>NP_000268.1:p.Arg408Trp	homozygous	592.6	96.2 ± 51.1

Abbreviations: Phe, Phenylalanine. Data are represented as mean±SD, or median (IQR), as appropiate
