# Supplementary material for: Evaluating Therapy and Growth in Children with Phenylketonuria: A Retrospective Longitudinal Study from Two Romanian Centers
Source: Medicina (Kaunas). 2024 Jul 22;60(7):1185. doi: 10.3390/medicina60071185 (PMC11279053; doi:10.3390/medicina60071185)
Supplement: Supplementary file 1 [file medicina-60-01185-s001.zip › medicina-3101802-supplementary/Supplementary Table S2.pdf]

Table S2. P-values for age-based comparison of growth parameters between PKU and control group

age (months)	WHZ	WAZ	HAZ	BAZ
0	0.696	**0.040**	**0.029**	0.192
3	0.126	**0.032**	0.443	**0.004**
6	0.735	0.290	0.325	0.929
9	**0.027**	**0.016**	0.274	0.440
12	**0.002**	**0.006**	0.762	0.150
15	0.138	**0.026**	0.681	0.297
18	**0.005**	**0.015**	0.559	**0.020**
21	**0.022**	**0.008**	0.589	**0.004**
24	**<0.001**	**0.022**	0.914	**0.002**
27	0.152	**0.041**	0.456	0.152
30	**0.033**	0.095	0.572	**0.038**
33	0.131	**0.006**	0.742	0.231
36	**0.048**	**0.015**	0.317	**0.095**

Abbreviations: WHZ, weight-for-height z-score; WAZ, weight-for-age z-score; HAZ, height-for-age z-score; BMI-for-age z-score. Statistically significant values are represented in bold.
